# Dr. Beck

**Published:** 1840-01

**Authors:** 


### CARL JOSEPH BECK, M D., PROFESSOR OF SURGERY, &c., IN FREIBURG.

C. J. Beck was the son of a country practitioner (Physicus), and was born at Gengersberg in the Kinzigthal, on the Rhine, on the 27th June, 1794. His father died before his birth, and his mother in 1799 entered into a second marriage with M. Schwörer, of Freiburg, to which place she removed with her two children. Young Beck was sent to the chief school at Freiburg, where he showed from the first great promise of excellence; he attended the university in the same place from the year 1808; here he remained until 1812, when he went to Tübingen, and spent a year pursuing his medical studies under Autenrieth, Kielmayer, &c. Immediately afterwards, while still only in his 19th year, on the march of the allied armies towards France, he was appointed to do the duty of a regimental surgeon in the field-hospital established for the benefit of the troops of Baden then blockading Strassburg. Here he had the great advantage of the counsel and assistance of the staff-surgeon-general, Meyer, and greatly distinguished himself in his treatment both of the sick and wounded. In consequence of his meritorious exertions, and after the proper examinations, he was promoted to the rank of regimental surgeon, and in this capacity made the campaign of Alsace, in 1815. The two following years, with the sanction of the government, and still retaining his military appointments, Beck spent in what may be called professional travelling, in company with his friend, Professor Chelius: they visited among other places Vienna, Berlin, Göttingen, Würtzburg, and Paris.

On his return in 1818, he was appointed professor-extraordinary and assistant-surgeon in the school of Freiburg, and at the same time took charge of the operative and ophthalmological branch of the surgical clinic. In 1819, he was named ordinary professor, taking for his share operative surgery, chirurgical nosology, diseases of the eye and ear, bandaging, &c. He subsequently taught medical jurisprudence, and occasionally assisted in the other departments. In 1828, he was named Counseller (Hofrath); in the following year, Privy Counseller (Geheimer Hofrath); in 1834, Höchstihr Geheimer Hofrath (whatever dignity that may be); and finally, in 1837, he received the crowning honour of a knight's-cross of the order of the Lion. Previously to this, however, in 1835, Professor Beck had begun to lose his health, and although he struggled manfully against his disease (an affection of the heart), returning, after intervals, to his professional duties, he finally sunk under it, on the 15th of June, 1838, leaving behind him a wife and four children. Dr. Beck was distinguished both as a practical surgeon and scientific teacher. The following are his principal works:—

Ueber die angeborene Verwachsung der Finger. Freiburg, 1819.—Ueber die Vorzüge der Lappenbildung bei der Amputation in der Continuität der Gliedmassen. Freiburg, 1819. Handbuch der Augenheilkunde. 2 Auflage, 1824-32.—Sacra semisæcularia, etc Drs. Prof. Menzinger: Insunt: im Animadversiones de capitis vulneribus practicæ, etc. Freiburg, 1833.—Die Krankheiten des Gehörorgans. Heidelbg. u. Leipzig, 1827.—Ueber den Kropf. Freiburg, 1833.—Abbildungen von Krankheitsformen aus dem Gebiete der Augenheilkunde. Heidelb. 1835.—Ueber die Anwendung der Ligatur, etc. ein Beitrag zur Therapie des traumatischen Blutungen. Leipzig, 1835.

